# Bicondylar tibial fractures: Internal or external fixation?

**DOI:** 10.4103/0019-5413.77130

**Published:** 2011

**Authors:** Gunasekaran Kumar, Nicholas Peterson, Badri Narayan

**Affiliations:** Department of Orthopedics and Trauma, Royal Liverpool University Hospital, Prescot Street, Liverpool, L7 8XP, UK

**Keywords:** Bicondylar fracture, circular external fixation, complications, internal fixation, minimally invasive, tibia, Ilizarov

## Abstract

Bicondylar fractures of the tibia, representing the Schatzker V and VI fractures represent a challenging problem. Any treatment protocol should aim at restoring articular congruity and the metaphyseo-diaphsyeal dissociation (MDD)—both of these are equally important to long-term outcome. Both internal and external fixations have their proponents, and each method of treatment is associated with its unique features and complications. We review the initial and definitive management of these injuries, and the advantages and disadvantages of each method of definitive fixation. We suggest the use of a protocol for definitive management, using either internal or external fixation as deemed appropriate. This protocol is based on the fracture configuration, local soft tissue status and patient condition. In a nutshell, if the fracture pattern and soft tissue status are amenable plate fixation (single or double) is performed, otherwise limited open reduction and articular surface reconstruction with screws and circular frame is performed.

## INTRODUCTION

**Figure 1:**
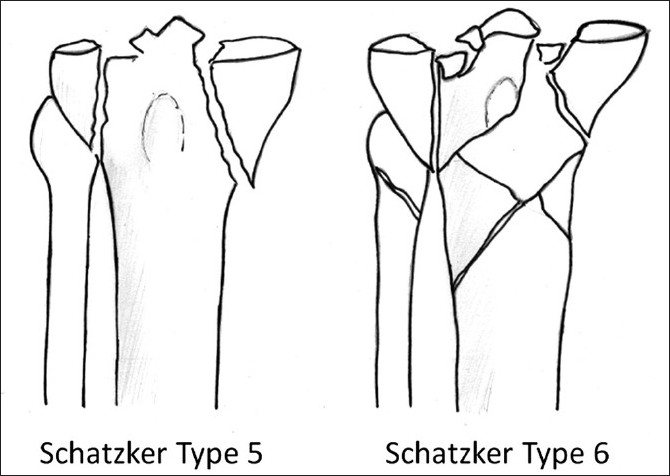
Schatzker classification of bicondylar fractures

Bicondylar fractures of the tibia, representing the Schatzker V and VI fractures, and largely part of the AO-OTA “C” type fractures [Arbeitsgemeinschaft für Osteosynthesefragen (German for Association for Study of Internal Fixation) – Orthopaedic Trauma Association] [Figure 1] present a bimodal spectrum of severity. They are often high-energy injuries in young patients, but are becoming increasingly prevalent in elderly patients, often consequent on low-energy falls. The goal of treatment of these injuries is reduction of the articular surface, and restoration of the metaphyseo-diaphyseal dissociation (MDD). The most important factor in deciding the timing and modality of definitive management is the status of local soft tissues.

In order to extract the relevant indexed publications regarding management of tibial bicondylar fractures, a PubMed search was performed using terms “tibia, bicondylar, plateau, Schatzker V, VI, external fixator, circular frame, minimally invasive, open fracture, complications”. Further searches were conducted on Medline via Health Informatics Resources (formerly known as National Library for Health) for the key words mentioned. This website has an advanced healthcare database search that allows Boolean operators and wildcard. It also allows combining results of key words searched. We analyzed the publications extracted with regard to their relevance to the preparation of this article. The publications deemed to be appropriate were assessed according to methods, materials, analytic methods, merits and drawbacks and the relevance of the conclusions and were cited appropriately.

## EVOLUTION OF TREATMENT

Non-operative treatment of bicondylar fractures, in the era before the use of internal fixation, yielded “acceptable results” as shown by Rasmussen in the early 1970s and in a 20-year follow-up by Lansinger **et al**.^1^ The main complications of non-operative management included stiffness and mal-union. With increasing patient expectations, and the recognition of complications of non-operative treatment, operative management assumed ascendancy. Traditional techniques of open reduction and rigid internal fixation of both condyles through a single anterior incision needed soft tissue stripping, and not unexpectedly, were associated with wound healing complications when adopted for all bicondylar fractures.^2^,^3^ Fine-wire external fixation as a means of reassembling the MDD gained popularity^4^,^5^ but it was recognized that this method was associated with its own unique complications such as the potential for septic arthritis of the knee due to intra-articular wire placement, and the risk of pin site infection. The introduction of modern plates with outriggers for insertion (such as the Less Invasive Skeletal Stabilization System – LISS) allows insertion of plates through smaller incisions, minimizing soft tissue damage. The capacity to use locking screws in these modern implants raises the feasibility of securely capturing the opposite condyle – a feature that can be exploited in fixing bicondylar fractures with a single lateral plate.^6^

## CONSIDERATIONS IN PREOPERATIVE PLANNING

Management of tibial bicondylar fractures is influenced by the patient’s condition as a whole, the surgeons’ assessment of the “fracture personality”, and most importantly, local soft tissue status.

Patient-related factors include age, co-morbidities, associated injuries, as well as the patient’s expectations of function following treatment. Salient aspects of the fracture personality that affect management and outcome are the degree of articular and metaphyseal comminution, bone loss, and the direction of the fracture lines. Soft tissue swelling can become significant enough to cause raised compartment pressures even in an apparently low-energy injury. In high-energy injuries, bruising and loss of skin integrity can take a few days to manifest even if there are no obvious skin wounds at presentation.

## INITIAL TREATMENT

Tibial bicondylar fractures typically occur in young patients with high-energy injuries and these patients should be assessed as per the ATLS^®^ protocol (Advanced Trauma Life Support, American College of Surgeons).^7^ Specific to tibial bicondylar fractures, assessment should include soft tissue status, distal neurovascular status, and continued monitoring for compartment syndrome.

Local damage control of a tibial bicondylar fracture is best achieved with stable spanning external fixation. This allows provisional reduction of the fracture, maintains fracture alignment and leg length, gives excellent pain control, helps in reduction of swelling, and allows for unhindered assessment and care of soft tissues. It also allows easy monitoring for compartment syndrome. Furthermore, it allows painless transport for patients to specialist trauma centers. Concerns about the possible risk of compartment syndrome due to acute restoration of length, and the increased risk of thrombo-embolic phenomena have proven unfounded.^8^,^9^ Spanning fixation does not cause long-term restriction of knee movement.^10^

A monolateral fixator, with two anteroposterior pins in the femur, and two anteroposterior pins in the tibia allows reasonably quick, easy and reproducible application. Care must be taken to avoid the zone of soft-tissue injury and bony injury – typically, the pins are placed at least 2 to 3 cm away from the furthest limit of bony injury visible on radiographs, and the same distance from visible soft-tissue injury. The knee joint is kept in about 15 degrees of flexion to allow non-weight-bearing mobilization of the extremity.

Additional pins, typically lateral to medial in the distal femur, and anteromedial to posterolateral in the tibia can be used for increased stability.^11^

## FURTHER INVESTIGATION

**Figure 2:**
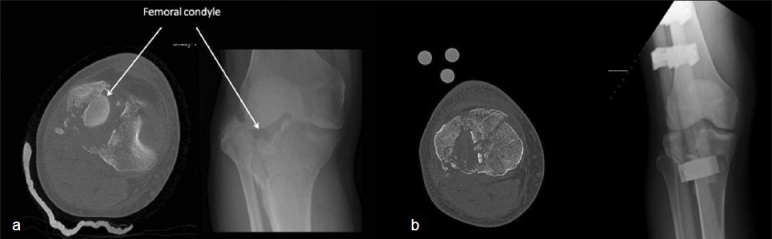
(a) CT scan before spanning external fixation - note the difficulty in interpretation of the CT due to overlapping femoral condyles. (b) CT scan after spanning external fixation - tibia is out to length and femoral condyle does not interfere with the interpretation of fracture configuration

Prior to availability of computed tomography (CT) scans, oblique radiographs of the knee joint complemented standard anteroposterior and lateral radiographs of the knee and proximal tibia. Presently, CT scanning, using 3-mm cuts is the investigation of choice for accurate assessment of the fracture. Scans should be performed only after application of spanning external fixation – this eliminates the possibility of multiple overlapping shadows, including an impacted femoral condyle interfering with interpretation of the scan [Figure 2]. The axial cuts are studied to understand the direction of the fracture lines, especially those of the medial condylar fragment. The sagittal and coronal reconstructions provide an estimation of metaphyseal ‘bone loss’, and the position of depressed articular fragments.

Mustonen **et al**., found a 36% incidence of meniscal injury, diagnosed by magnetic resonance imaging (MRI), in tibial plateau fractures but could not find any correlation with fracture type or amount of fracture depression.^12^ Mui **et al**., have shown that CT scans have a high specificity with regards to ligamentous injuries in tibial plateau fractures but are poor in assessing meniscal injuries.^13^ MRI scan could over-diagnose meniscal injuries. Meniscal injuries detected do not always require surgery as often these injuries are just contusions.^14^

While MRI has been shown to be sensitive in identifying meniscal and ligamentous injury in tibial condylar fractures, it is of less importance in complex bicondylar fractures than in the simpler fracture patterns.

## DEFINITIVE TREATMENT

It is not unusual for soft tissue swelling to take up to two weeks to settle enough to permit invasive surgery. As stated earlier, the use of spanning external fixation allows patient mobilization during this period, and allows for unhindered monitoring of the status of the skin.

The two broad aspects of definitive treatment are 1) restoration of the articular surface and 2) restoration of the MDD. Irrespective of the technique employed, the principles remain the same as with any other fracture surgery. The articular block is first anatomically reduced and rigidly stabilized. The metaphyseo-diaphyseal part of the fracture is dealt with by indirect reduction, providing relative stability whilst ensuring satisfactory length, alignment and rotation. This is achieved either using plate fixation or circular external fixation.

### Restoration of the articular surface

Restoration of the articular surface can be accomplished by closed means in the simpler fracture patterns. However, higher energy fractures are usually associated with depressed articular fragments, and need an open reduction of the lateral condylar articular surface and screw fixation. Indirect articular reduction could be achieved by ligamentotaxis using a femoral distractor applied such that it does not interfere with intraoperative fluoroscopy and surgical field. Arthroscopy-assisted fracture reduction is useful in simpler fractures, such as partial articular fractures,^15^ but fluid leakage into compartments and possibility of compartment syndrome often precludes it in higher energy fractures. There is, however, potential advantage of assessing the intra-articular structures and treating any meniscal injuries.

The anatomy of the fracture is such that the vast majority is associated with depression of the articular surface of the lateral condyle. Depressed fragments cannot be elevated by closed reduction. Some of them can be elevated by making a bone window percutaneously, and tapping with a bone impactor. The majority however need an open reduction. Direct visualization of the articular surface can be achieved by performance of a submeniscal arthrotomy, and elevating the meniscus after dividing the coronary ligament. The depressed fragments are gently disimpacted, and elevated to the level of the articular surface. The fragments are temporarily held in place with K-wires, and substituted with screws. 6.5 mm screws afford excellent grip, but there is a vogue now to use multiple smaller screws in a “raft” configuration – these are 3.5-mm or 4-mm screws, placed subchondrally. There is biomechanical evidence to suggest that a “raft” of these screws affords as good a grip as the larger screws, particularly in osteoporotic bone.^16^,^17^

Bicondylar tibial plateau fractures often have bone defects due to compression of the cancellous subchondral bone. After reduction of the articular fragments, options for filling the metaphyseal bone defects include iliac crest autograft, allograft and bone substitutes such as calcium phosphate and hydroxyapatite. A randomized trial has shown calcium phosphate to have reduced subsidence rates in comparison with autograft.^18^ Iliac crest autograft however continues to be the ‘gold standard’ and has the potential advantage of providing osteogenic, osteoinductive and osteoconductive properties, at the potential expense of donor site morbidity.

After the articular surface has been reconstructed, metaphyseal defects filled, and the fracture stabilized, the meniscus is reattached using strong absorbable sutures, in effect completing a red-on-red repair. The capsule and iliotibial band are closed, thus completing the articular reconstruction. Attention is then turned to restoration of the MDD, either with internal or external fixation.

### Restoration of the metaphyseo-diaphyseal dissociation

#### a. Plate fixation

**Double plating using one incision**: Double plate fixation through a single midline incision was common in the early days of internal fixation. However, this procedure involves significant soft tissue stripping leading to an unacceptably high incidence of infection and non-union,^19^ and is hence not preferred now.

**Single lateral locking plate**: Biomechanical studies suggest a clinically insignificant difference in collapse of the medial condyle following fixation of experimental bicondylar fractures with a single lateral locking plate.^20^ However, the direction of the fracture line in the medial condyle can vary.^21^,^22^ A predominantly “back to front” medial condylar fracture can be gripped adequately with locked screws from a lateral plate.

**Figure 3:**
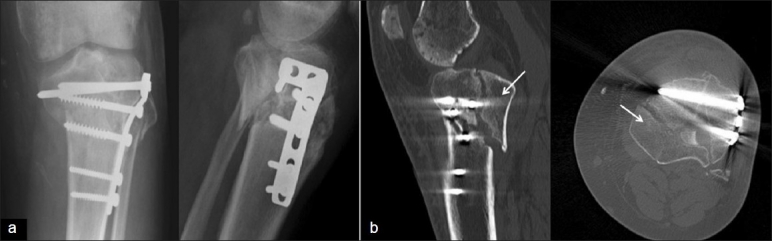
(a) Catastrophic failure of single-plate fixation as the screws have completely missed the coronally based medial condylar fragment. (b) CT scans showing the medial tibial condylar fragment (white arrows) is not stabilized

However, lateral to medial screws from a lateral locked plate cannot provide adequate purchase on an oblique fracture, or a predominantly posteromedial fracture. The literature suggests failure in restoring and maintaining alignment in about 8–13% of patients of bicondylar fractures treated with single lateral locked plates.^6^,^23^ Therefore, the use of a single lateral locking plate in all bicondylar fractures is likely to result in failure in certain fracture patterns due to inadequate grip of the medial fragment [Figure 3a and b].

**Figure 4:**
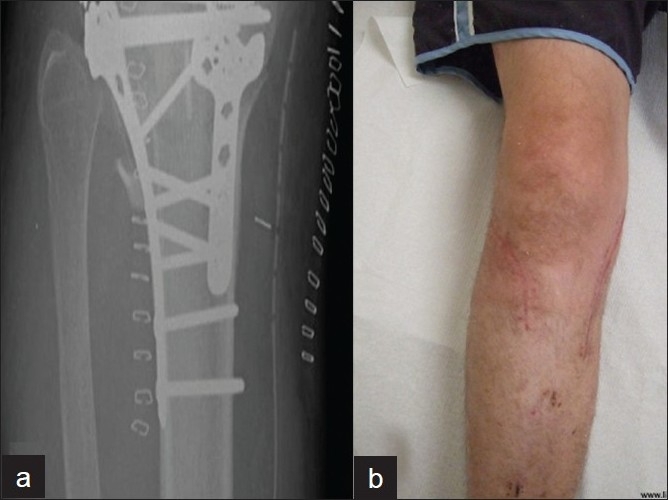
(a) Buttress plate fixation of a large posteromedial fracture. (b) Clinical photograph of patient with fixation in (a), showing typical posteromedial and anterolateral incisions

**Double plates with two incisions**: Due to complications of a midline incision to stabilize both condyles, a double-incision approach has been used. The medial condylar fragment is stabilized first, using a posteromedial approach.^24^ A one-third tubular plate, in antiglide mode, usually suffices in patients with good bone quality. Newer precontoured locked plates, specially designed for the posteromedial surface of the proximal tibia can also be used [Figure 4a].

**Figure 5:**
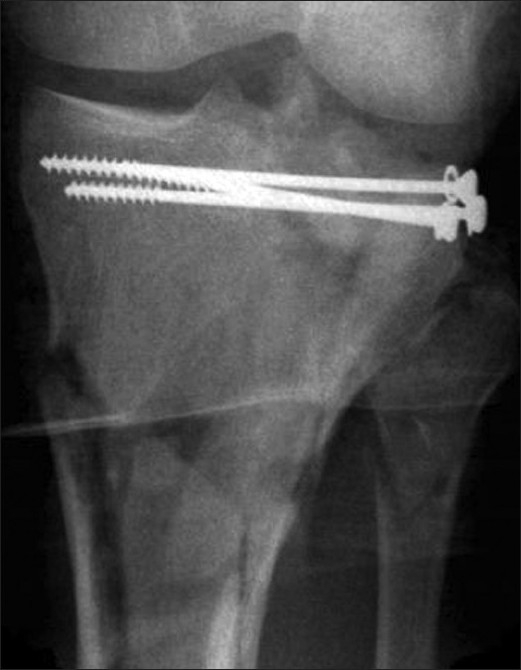
Raft of screws for stabilization of articular block

**Figure 6:**
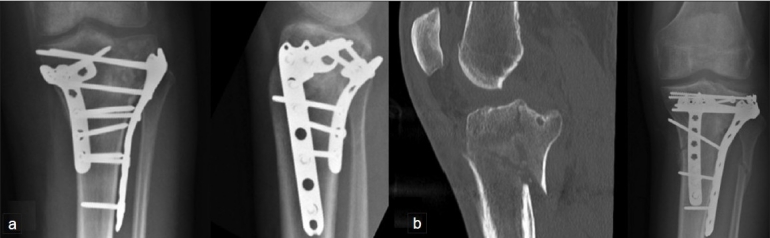
(a) Posteromedial plate and lateral plate fixation of a bicondylar fracture. (b) Sagittal CT scan showing the apex of the posteromedial fragment which was stabilized with a posterior plate followed by lateral plate stabilization

The lateral condylar fracture is then approached through a separate anterolateral incision – the preservation of the anterior skin bridge results in a negligible risk of wound breakdown [Figure 4b]. Following restoration of the articular surface through open reduction and “raft” fixation [Figure 5], a lateral condylar plate is used to restore the MDD [Figure 6a].

While this approach seems intuitively simple, affording reduction of both fractures and preservation of soft tissue integrity, it is not free of complications. Published literature suggests a deep infection risk of about 8%, malalignment in the coronal plane in about 8%, and sagittal plane malalignment in about 20%.^25^

**Figure 7:**
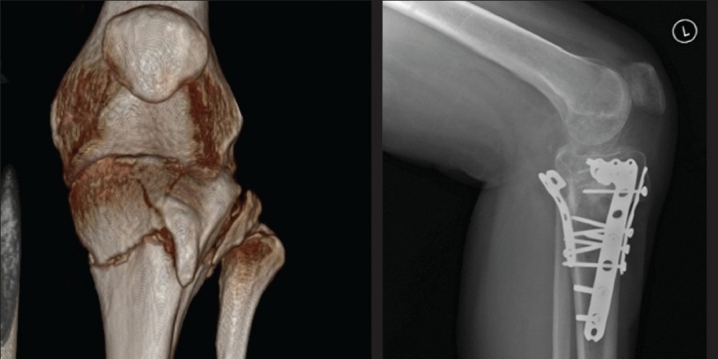
3D CT reconstruction showing tibial tuberosity fracture. X-ray (lateral view) of knee joint with proximal half of tibia showing sequence of stabilization of this fracture - posteromedial plate, tibial tuberosity fragment stabilized with anterior plate and lateral plate

**Plating in unusual fracture patterns**: A study of the axial CT can sometimes reveal unusual fracture patterns, usually posterior or posterolateral. Specific approaches, directed to neutralize the fragment, are needed in these cases. In the presence of a large posterolateral fragment, a posterior approach to the knee provides adequate access to achieve fracture reduction and plate fixation in buttress mode [Figure 6b]. A variant of the posteromedial approach can be used for posteromedial fracture lines.^26^ Similarly, an associated fracture of the tibial tuberosity can be stabilized with a plate and screws [Figure 7].

#### b. Circular frame with or without limited open reduction and screw fixation

**Figure 8:**
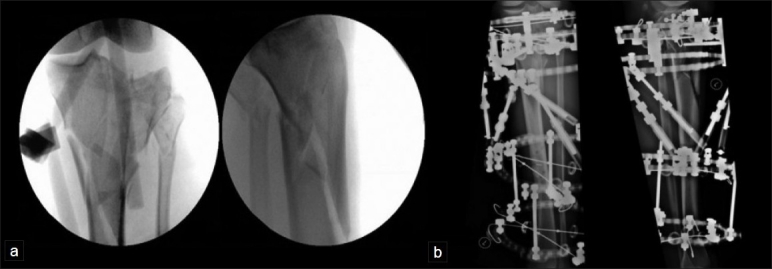
(a) Preoperative image intensifier pictures (antero posterior and lateral view) showing bicondylar fracture with significant comminution at the metaphyseo-diaphyseal area. (b) Same fracture stabilized with a circular frame (Taylor Spatial Frame^®^) after open reduction of the articular surface (^®^ Smith and Nephew, Memphis, Tennessee)

A fine-wire circular fixator allows noninvasive reconstitution of the MDD [Figure 8a and b]. The technique, in experienced hands, is straightforward. The completely noninvasive nature of the procedure obviates any risk of wound breakdown. Wedge fragments of bone can be repositioned using olive wires. The security of fixation may allow earlier weight-bearing and its attendant benefits. The literature also suggests that such forms of treatment may be more beneficial for comminuted fractures.^27^

Circular fixators such as an Ilizarov frame or a Taylor Spatial Frame can be repeatedly adjusted during the course of fracture healing in order to reduce gaps, and to correct the slight but significant collapse that often occurs at the metaphyseal level during fracture healing. This last feature, the capacity to fine-tune reduction of the metaphysis is probably the most important facet of treatment with a circular external fixator. In addition, there is no “footprint” on the tibia following removal of the fixator, allowing easy performance of secondary surgery should this be necessary.

However, external fixators are a definite encumbrance to the patient. There is also a definite risk of pin-site problems,^28^ though this can be reduced significantly with careful pin handling, careful techniques of pin insertion and postoperative care. Pin-site infection in very proximal tibial locations can lead to septic arthritis, and it is generally recommended that the most proximal pin in the tibia should be no higher 12-15 mm from the level of the knee joint.^29^

This carries implications in the treatment of very proximal bicondylar fractures with circular external fixation - the size of the proximal fragment may mean that some of the wires in the proximal fragment are definitely intra-articular, increasing the risk of joint sepsis. A transfibular wire adds to the stability of the frame. There is potential for increased risk of septic arthritis as the proximal tibiofibular joint can communicate with the knee joint. In addition, very proximal dissociations (classically above the tibial tuberosity) often need an extension of the circular frame across the knee joint, with its attendant morbidity.

A multicentre randomized controlled trial by the Canadian Orthopedic Trauma Society showed that the advantages of circular frame included lower infection rates, less intraoperative blood loss, and a shorter hospital stay in comparison with plate fixation. Furthermore, the number of unplanned repeat surgical interventions, and their severity, was greater in the open reduction and internal fixation group. Functional outcomes were however similar to plate fixation patients at two years. Due to the higher number and severity of complications in open reduction and internal fixation, the authors were in favor of circular external fixation for these difficult-to-treat fractures.^30^

### Management of associated local injuries

#### a. Meniscal and ligamentous injuries

Tibial bicondylar fractures are often associated with significant soft tissue swelling precluding arthroscopic or open repair of soft tissues. As discussed earlier, MRI scans of tibial bicondylar fractures show high incidence of meniscal or ligamentous injuries. However, all meniscal injuries do not require intervention.^14^ If lateral plate stabilization is planned, lateral meniscal tears could be repaired at the same time. Collateral and cruciate ligament injuries can be treated non-operatively initially. Symptoms and functional requirements can be reassessed and dealt with after stabilization of the fractures.

#### b. Compartment syndrome

Due to the presence of significant soft tissue injuries patient should be frequently assessed for compartment syndrome both pre and post surgery. If compartment syndrome occurs prior to surgical intervention, then, release of compartments and spanning external fixation is performed. This often precludes plate fixation but definitive stabilization of fracture should be decided on fracture pattern, soft tissue status and potential for skin cover. If compartment syndrome occurs after plate stabilization, then, compartments are released and early cover of the implants may require plastic surgical care.

#### c. Vascular injuries

Arterial injuries are uncommon. However, when it occurs it is an emergency, requires emergency vascular repair and spanning external fixation, with this being applied in such a way that it does not interfere with positioning the patient prone. If the tibial plateau is the only injury in the limb, then arteriography in radiology suite is not essential as, in high energy tibial bicondylar injuries popliteal artery is damaged between adductor hiatus and popliteal artery trifurcation. This is due to relative immobility of popliteal artery between these two points. On-table arteriography can be performed in case of any doubt or in the presence of other associated ipsilateral limb injuries. Options for definitive treatment of fracture are then limited due to the presence of associated prophylactic fasciotomy wounds.

### Outcomes following bicondylar fractures

**Figure 9:**
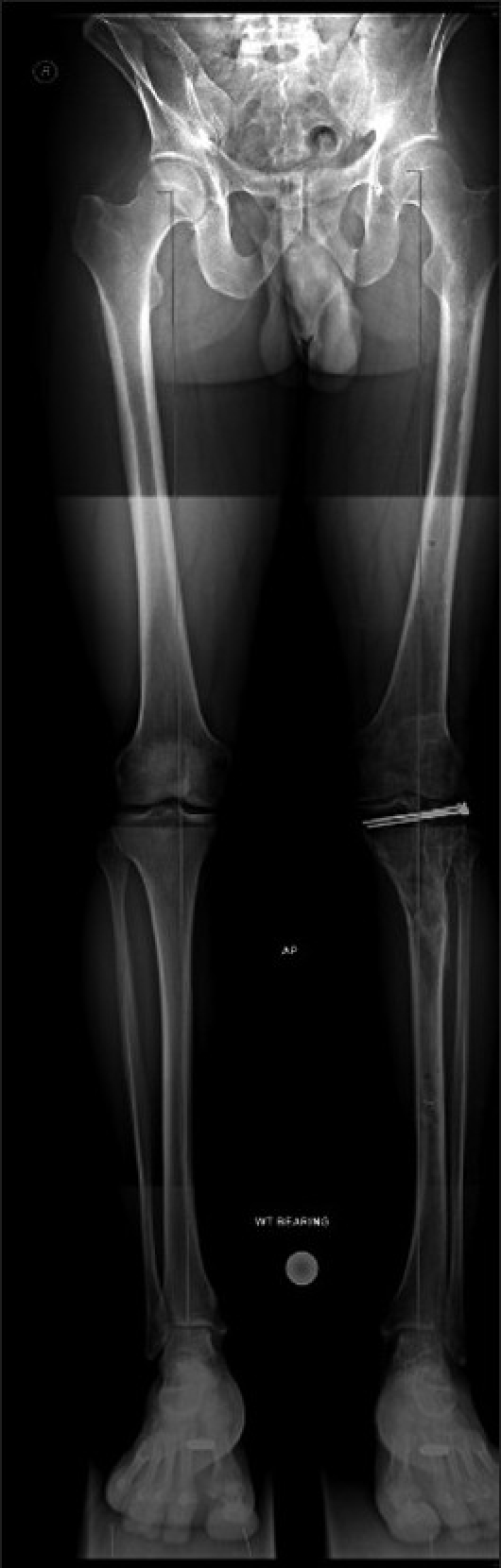
Long leg films (same case as in Figure 8) showing a healed fracture with good restoration of the mechanical axis of the limb

Ideal outcome assessment would include prospective collection of data, postoperative CT scans to quantify joint incongruity and fracture reduction, long leg standing views [Figure 9] to assess mechanical axis deviation, and limb length discrepancy, as well as long-term follow-up to assess clinical and radiological features of secondary osteoarthritis. Outcomes should also include the long-term results of arthroplasty. There are no studies that have measured all these outcomes.

Honkonen described poor results associated with a varus malalignment of greater than 5 degrees, a valgus tilt of greater than 10 degrees, articular displacement of greater than 4 mm and condylar widening of greater than 10 mm.^31^

Following a symposium of the American Orthopedic Association, Marsh **et al**.,^32^ reviewed the literature on articular reduction and clinical outcome of tibial plateau fractures. Functional outcomes appeared to be related more to stability of the knee joint and maintenance of alignment of the metaphysis. They concluded that there was little rationale to support the assertion that an articular step of greater than 2 mm was associated with poor outcome.^32^

### Our protocol

All patients in our unit are investigated with CT scan after application of spanning external fixator, if required. We do not perform MRI investigation acutely. The protocol for the definitive management of bicondylar fractures is based on the assessment of the MDD, and the pattern of the fracture. We use circular external fixation, or plating as appropriate, based on the inherent advantages and disadvantages of each. If an associated lateral meniscal injury is identified at open reduction, it is dealt with as indicated. However, we assess the patients after they have recovered from their bony injury, to decide whether any meniscal or ligamentous injuries are present and causing functional problems.

Restoration of the articular surface, using either closed or open methods is common to both methods of treatment. Our choice of articular surface reduction is indirect with traction/ligamentotaxis and fluoroscopy control and, more commonly under direct vision under direct vision. We do not perform arthroscopy-assisted articular fracture reduction for bicondylar fractures.

Based on the fracture pattern and communition we decide whether stable internal fixation is possible. If stable internal fixation is possible and local soft tissue status permits, then, internal fixation is carried out. We have not had the need to perform additional external fixation and generally do not recommend it as it adds to the complications of both procedures – soft tissue stripping and knee stiffness.

We recognize that a circular external fixator has a higher risk of significant problems with pin-site infection if the wires are intra-articular, i.e., within 12 mm to 15 mm of the knee joint. Such a situation is likely to arise in supra-tubercular dissociations, which often need a cross-knee extension to a circular frame, with its attendant problems. The most obvious advantage of a circular frame, its capacity to fine-tune alignment of the tibia, is in cases with significant comminution at the metaphyseo-diaphyseal junction.

Plate fixation has significant benefits in terms of decreased number of outpatient visits, the avoidance of pin-site problems, and the morbidity, albeit temporary of any external fixator device. However, we also note that plate fixation has the risk of malalignment, now recognized to be one of the most important predictors of outcome of these fractures.^24^ We believe that there is an increased scope for malalignment when plating comminuted fractures, which can also collapse during healing.

Fractures that are comminuted at the metaphyseo-diaphsyeal junction are also likely to be associated with poor overlying soft tissues, suggesting that a circular external fixator may be associated with lesser risks of wound breakdown. In cases suitable for plate fixation, we study the morphology of the medial condylar fragment - cases with a predominantly sagittal fracture line and minimal medial condylar comminution are treated with a single lateral locked plate.

**Figure 10:**
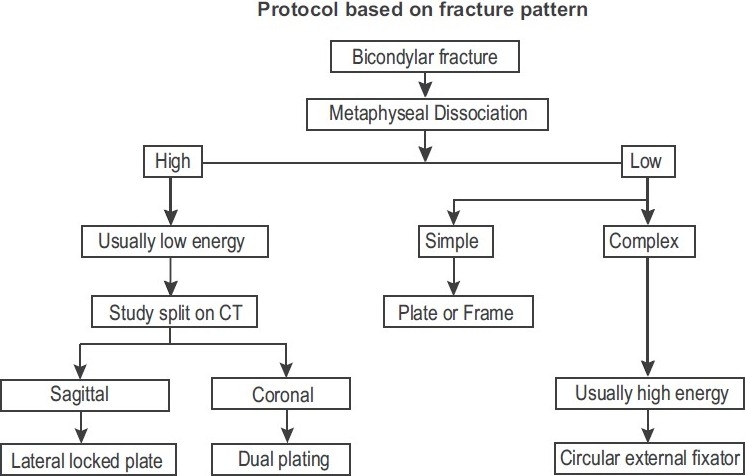
Protocol followed in the authors’ institution for managing tibial bicondylar fractures

Double plating, using two separate approaches is used for those injuries where the medial condylar fracture is more coronal than sagittal. The medial condylar fracture, usually simpler in configuration, is reduced and fixed first, thus, effectively converting the injury to a partial articular fracture. The lateral condyle is then reassembled to the rest of the tibia using a separate plate. This protocol is summarized in Figure 10.

## CONCLUSION

Bicondylar fractures are heterogeneous injuries, with a high risk of complications of treatment. The treating surgical team should have in their armamentarium the capacity to treat these fractures with either internal or external fixation, depending on the nature of injuries. Whatever method of stabilization is chosen, the principles of stabilizing these high-energy injuries are soft tissue care, accurate articular surface reduction and maintenance, whilst achieving satisfactory length, rotation and alignment. The choice of treatment should be dictated by the soft tissues and fracture configuration.
